# Downscaling Climate Models: Sharpening the Focus on Local-Level Changes

**DOI:** 10.1289/ehp.120-a22

**Published:** 2012-01-01

**Authors:** Catherine M. Cooney

**Affiliations:** Catherine M. Cooney is a science writer living in Washington, DC.


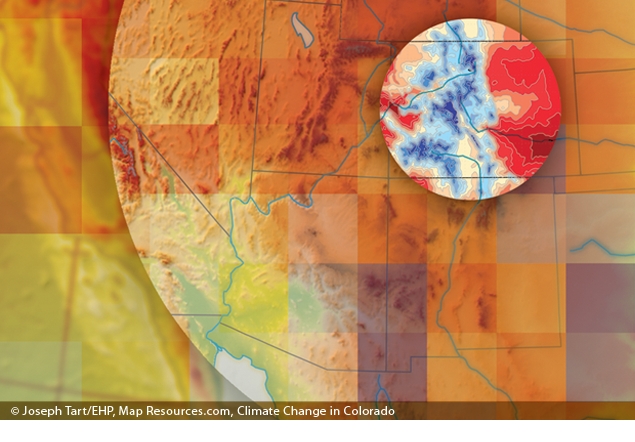
Most climate change projections are developed using global-scale models that generate average temperature changes that can be expected to occur over decades and far into the future. These global models are unable to represent granular atmospheric features such as cloud cover, airborne particles, and local pollution sources. Yet these smaller details can have a big impact on local climate, which is one reason the effects of climate change are expected to vary depending on geographic location.^1,2^

Downscaled regional climate models (RCMs) provide grist for climate change adaptation planning at the local and regional level.

© 2012 Joseph Tart/EHP; Map Resources; Ray et al.^26^ This graphic is for illustration purposes only and does not represent an actual RCM.

“Downscaling” climate models are an attempt to bridge the gap between global and local effects by layering local-level data over larger-scale climate models. Downscaled modeling examines relatively small areas in detail—in some cases down to 25 square kilometers,^3^ a far higher resolution than that offered by global climate model simulations. The goal is to generate more locally relevant projections of long-term weather patterns for regions, states, and cities.

In 2000, in one of the first projects to apply downscaling to simulate local effects, a small group of researchers designed and tested a system to estimate the health impacts of climate change on individuals in a specific location: New York City. Their analysis, developed under the banner of the New York Climate and Health Project (NYCHP),^4^ incorporated local heat and air-quality data as well as land-use data such as new development and roads, since those also impact surface temperature and air quality. Using these data, the team projected that higher-than-normal temperatures and resulting increases in ground-level ozone production brought on by climate change could result in a 4.5% increase in ozone-related deaths across the metropolitan area by the 2050s.^5^ They also calculated that summer heat–related mortality across the area might increase 70% on average over the same period.^6^

“Downscaling work provides a view of how climate change may impact health in the future, it begins to describe for us the range of possible answers to some of the public health questions that we have today, and it gives us a sense of different possible alternative futures,” says Kim Knowlton, a senior scientist with the Natural Resources Defense Council, who worked on the NYCHP. “At this point, downscaled modeling results are invaluable to adaptation planning, and the expertise in the modeling community is improving all the time.”

Thorsten Wagener, an associate professor at The Pennsylvania State University who specializes in hydrology, says there is not a major research university in the United States that doesn’t have someone working on downscaled regional climate modeling (RCM) because the impact of climate change for planners and managers is a crucial question at the moment. Wagener says, “Once the IPCC [Intergovernmental Panel on Climate Change^7^] used models to project climate change in the future, scientists quickly moved into asking, ‘What does global warming actually mean for a person in the street, for the power supply, for our economy, for our health, for all sorts of things?’” In a few cases public health officials and resource managers have already begun using these downscaled data to develop climate-change adaptation plans.^8^

## Global Climate Models: Unpacking the Black Box

By its nature, climate is both a chaotic and extraordinarily complex phenomenon with fluctuations that result naturally from interactions between the ocean, atmosphere, land, cryosphere (frozen portion of the Earth’s surface), and changes in the Earth’s energy balance resulting from volcanic eruptions, variations in the sun’s intensity,^9^ and alterations in atmospheric composition that alter the balance of incoming and outgoing solar energy. The original global climate models, known as general circulation models (GCMs), simulated the interactions between the oceans and the atmosphere.

**Figure:**
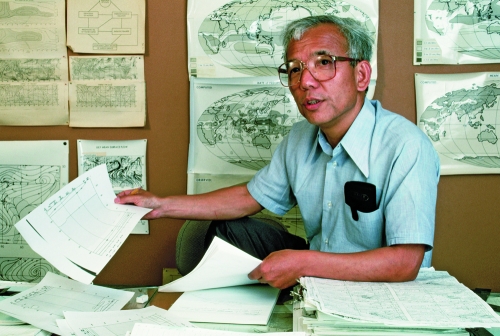
Results from the world’s first ocean–atmosphere GCM were published in 1969 by Syukuro Manabe (pictured at left) and Kirk Bryan, two scientists with the Geophysical Fluid Dynamics Laboratory at Princeton University.^27^ The two used a series of mathematical models to predict weather patterns over a few days. Their “coupled GCM” (“coupled” because the model connected ocean and atmospheric models so they could interact as these systems do in nature) set the stage for an entirely new way to conduct research and opened the door to understanding the highly complicated world of natural climate processes. © 2012 Jim Sugar/Corbis

Since the 1990s climate scientists have made great leaps in their understanding of and ability to describe previously undescribed environmental processes, such as how sunlight affects ice floating in the Arctic or how oceans absorb carbon dioxide. Newer climate models incorporate equations that estimate how these environmental processes affect temperature.

Simply stated, a global climate model is a three-dimensional grid of boxes representing 150- to 200-square-kilometer blocks of Earth. The boxes are stacked vertically and horizontally and cover the globe. Each box describes the wind movement, rainfall, temperature, and other characteristics for that specific block. Modelers then apply well-established principles of physics to estimate, for example, how winds and rainfall move through each box and alter the winds and rainfall in nearby boxes. The model analyzes and combines the data calculated from each box to generate a larger picture of how the Earth’s climate might change.^10^

All global climate models are essentially GCMs because they simulate changes in winds, temperatures, and atmospheric pressures simultaneously over the entire globe. In addition, many climatologists study much simpler intermediate complexity models that illustrate fundamental environmental processes, such as atmospheric dynamics. Many climate researchers use intermediate complexity models to test the accuracy of larger, more detailed models that estimate climate changes far into the future, says David Pierce, a programmer/analyst in the division of climate, atmospheric science, and physical oceanography at Scripps Institution of Oceanography.

Some 15–20 institutions worldwide^11^ maintain large GCMs, many of them government- or university-sponsored, says Gregory M. Flato, chief of the Canadian Centre for Climate Modeling and Analysis at Environment Canada. Fundamentally, each model is trying to simulate the same thing, although the models differ in their particular specifications and formats. The researchers in each modeling group apply their own scientific judgment in approximating the many physical processes relevant to climate, Flato says.

In many ways global climate modeling is as much an art as it is a science, adds Spencer Weart, director emeritus of the Center for History of Physics of the American Institute of Physics. “Global climate models are as different from each other as people,” Weart says.

“If we were able to perfectly represent every process that is relevant, there would be no need for approximations and no need for estimating smaller processes on a larger scale,” explains Claudia Tebaldi, a research scientist at Climate Central^12^ and adjunct professor at the University of the British of Columbia. “We are actually happy that there are more modeling centers that do these things in isolation because every answer is a legitimate one. We look at the many responses [from different modeling centers], and the reliability of the projections is enhanced when we see that the models are giving the same general answer.” At the same time, if the models provide different answers in a particular area, “it tells us that our understanding needs to improve,” she says.

**Figure:**
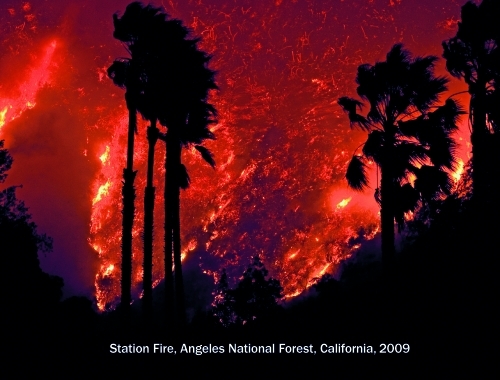
Modeling centers archive their results, and many are contributed to the World Climate Research Programme,^28^ a network comprising partners such as the International Geosphere–Biosphere Programme and the International Human Dimensions Programme on Global Environmental Change. The model results are freely available online for analysis. The same centers are working on RCMs, and most share these data with researchers around the globe. A few states have already made relevant RCM data available to the public. California, for example, recently unveiled Cal-Adapt, a website produced by the state’s science and research community that describes potential changes in wildfire activity, sea-level rise, snowpack, and temperature, all downscaled to the state’s geography.^29^ All users of Cal-Adapt have access to interactive maps—some linked to census tract data—and original source data. © 2012 Aaron Huey/National Geographic Stock

Viewing the Earth as simulated by a global climate model is similar to looking at a blurry photograph because of its coarse resolution. For example, one might recognize the general shape of the U.S. West Coast, but geographic details such as the inlets and bays along the Pacific Northwest coast are missing. Downscaling techniques incorporate these specific geographic details into a model. The inclusion of the shape of local water bodies or a mountain range’s higher and lower elevations creates a model that can simulate the wind speed, up- and downslope flows, evaporation, and other weather-related processes that affect the local environment, Tebaldi says.

## What Does Downscaling Show Us?

Regional modelers use different approaches to downscaling. One approach is dynamical downscaling, which feeds data output from global climate models into regional meteorological models to simulate local weather conditions. “For dynamical downscaling, you are basically nesting a higher-resolution regional model into a global climate model,” says Bill Kuo a senior scientist at the National Center for Atmospheric Research (NCAR). The global model can provide the large-scale changes, and the regional model provides the regional climate changes (ie., in temperature and precipitation) in much greater details because the local topography are much better resolved by the regional model.

But the level of detail involved when using the dynamical technique strains computer capabilities, so computations can only tackle outputs from individual climate models and short “time slices” (typically 3–5 years, Knowlton says).^13^ This makes it virtually impossible to conduct multicentury-long simulations for local conditions the same way that coarse-resolution global climate models are run.^14^

A second approach is statistical downscaling, which uses a series of equations to convert global-scale model output to regional-scale conditions. The underlying concept is that local climate is conditioned by large-scale climate and by local physiographical features such as topography and vegetation. Statistical downscaling requires identifying empirical links between large-scale patterns of climate elements and local climate. Once such links are built, then, statistical downscaling can be used to infer local climate changes using output from global or regional models, Kuo says. Researchers can downscale emissions scenarios for many models and many decades or even centuries with this approach because the statistical approach requires less computational effort than dynamical downscaling.^15^

One way climate scientists are answering the ubiquitous question “how does climate change affect me?” is by focusing individual RCMs on particular health-related issues such as heat waves or drought. For example, Noah S. Diffenbaugh, an assistant professor of environmental earth system science at Stanford University, and his colleagues recently reported that intense heat waves equal to the longest on record from 1951 to 1999 are likely to occur up to 5 times between 2020 and 2029 over areas of the western and central United States.^3^ This study analyzed geographic quadrants measuring 25 square kilometers, and it was unique in that the authors ran 25-kilometer simulations for multiple decades, multiple times to capture the internal variability in the climate system. “No one has ever completed this kind of climate analysis at such a high resolution,” Diffenbaugh says.

In another first, researchers at NCAR and the University of Kansas are developing climate models that incorporate the urban heat island effect (i.e., cities are warmer than surrounding rural areas because of factors such as greater air pollution and the effects of concrete on heat retention). Most global climate models don’t account for urban surfaces^16^ even though more than 50% of the world’s population lives in a city or metropolitan area.^17^ However, there are signiﬁcant differences in energy balance, temperature, humidity, and storm runoff between urban areas and rural surfaces.^18^

**Figure:**
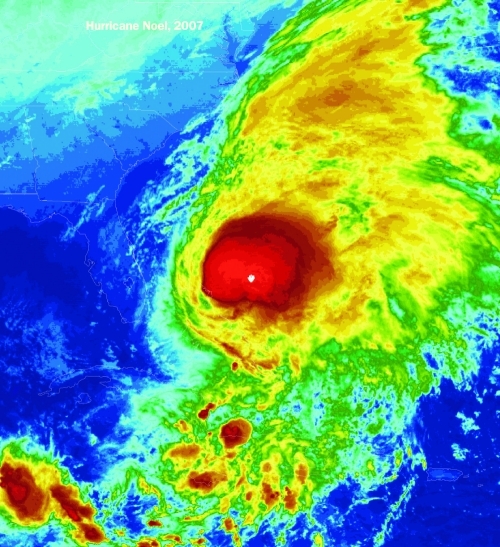
RCMs are being used to simulate extreme weather events such as heavy rains, droughts, and hurricanes, which occur at the local or regional level and thus are difficult for global climate models to recreate.^30^ Regional downscaling can simulate extreme weather events on the smaller scale because researchers input environmental processes (e.g., wind flow, rainfall) that are specific to that particular area. Global climate models, on the other hand, are able to simulate extreme weather events, but because their simulations are global, the resolution is coarse, and the extreme event often appears less powerful than those happening in real time, says David Pierce of the Scripps Institution of Oceanography. In other advances, climate researchers and modelers are beginning to address changes over the time scale of a few years to a couple of decades, says Claudia Tebaldi of the University of the British of Columbia. If successful, this type of effort would provide important information for local adaptation decisions. The upcoming Fifth Assessment of the Intergovernmental Panel on Climate Change will include an assessment of these new types of climate change simulations. © NOAA

Public health scientists are continuing to analyze the NYCHP’s downscaled data.^19^ Recently, Knowlton and colleagues, including study leader Perry Sheffield, an assistant professor of preventive medicine at Mount Sinai School of Medicine, estimated future pediatric asthma emergency department visits associated with ground-level ozone changes across the New York City metropolitan area, comparing actual visits in the 1990s with projected visits in the 2020s. They estimated that climate change could cause a 7.3% increase in regional summer ozone–related asthma emergency department visits by the 2020s. When population growth was factored in, the projections of morbidity related to ozone were even larger.^20^

In other work, RCMs are being used to simulate the spread of infectious disease. Penn State researchers Matthew Thomas and Michael Mann are collaborating on a model that uses local day-to-day temperature and precipitation conditions to better understand how these conditions influence the spread of malaria and dengue, the two most significant vectorborne diseases worldwide. Until recently researchers have relied on seasonal temperature averages occurring year to year, Mann says. But for malaria, he says, it’s important to understand how temperature changes from hour to hour throughout the day, because the incubation period of the parasite that causes the disease is exquisitely temperature sensitive.

Downscaling climate models are not restricted to wealthy countries. Scientists in developing countries are also beginning to simulate local climate change impacts using statistical downscaling. The Regional Climate Research Network^21^ has an RCM originally developed by NCAR that operates on a variety of computer platforms and encourages collaboration between “South–South” and “North–South” scientists.^22^ “I think most of the truly developing countries are not doing their own climate modeling,” Kuo says. “Instead most of the developing counties are relying on the projections done by the industrialized countries, and then they are doing their own statistical interpretation.”

Much of what can be done with global climate models depends on computational capabilities, and those capabilities are continuing to grow, modelers say. With increased computer power, global models will move toward achieving higher resolution and eventually produce local simulations similar to those currently produced by RCMs, says Johannes Feddema, a professor in the Department of Geography at the University of Kansas. “In the next 30 years I think there will be a mergence of global climate models and high-resolution modeling,” Feddema says. “Global models will run [at] one-kilometer [scales] in twenty to thirty years, which is the same resolution of today’s high resolution regional models.” But the global models have a long way to go before they are able to simulate regional conditions with the same clarity as RCMs.

## Clarifying Uncertainty

Water managers, particularly those in the western United States, have been ahead of the pack in employing RCMs to predict future water needs in the region. Yet many modelers also understand that regional data can go only so far in providing information for decision makers facing an uncertain climate. Brad Udall, director of Western Water Assessment, one of several Regional Integrated Sciences and Assessments programs funded by the National Oceanic and Atmospheric Administration, says that RCMs conducted on the Colorado River Basin can reliably predict only up to 8 years or so into the future. Water managers usually must plan 50–100 years in the future when considering new infrastructure, he says.

Linda Mearns, executive director of the North American Regional Climate Change Assessment Program, agrees that RCMs are just one piece of the adaptation puzzle for a world already facing increasing climate variability. She says more regional modeling is not necessarily the most important tool for adapting to climate change. Uncertainty is going to be a part of climate change planning regardless of how small the area a climate modeler can describe or how detailed the results from an RCM. The important point, Mearns says, is to recognize those uncertainties and figure out how to make decisions based on them: “There is a whole other component [of adaptation planning] that deals with making decisions for a particular resource area even though there are a lot of uncertainties.”

**Figure:**
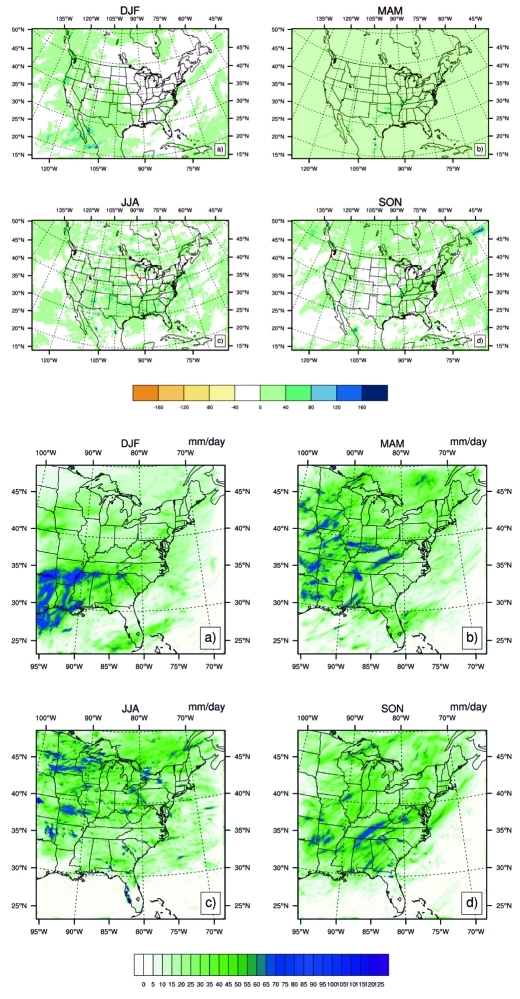
Two different climate models project seasonal averages of highest 1-day precipitation in 2030. A comparison of output from the global-scale Community Climate System Model 3 (top) and the regional-scale RegCM3 (bottom) illustrates the finer grain of detail made available with downscaling. © 2012 Noblis, Inc.

All climate models are used to make predictions based on different assumptions. Researchers input assumptions that flesh out any environmental processes that aren’t fully explained by science, such as the impact of soot on weather processes. They also input assumptions about the future level of greenhouse gas emissions. These assumptions add to the uncertainty inherent in climate models, which Wagener says is compounded with downscaling, as uncertainty cascades through each modeling stage. “With every step there are uncertainties that are naturally being added because we have to use a different set of assumptions for each new model that we add,” he explains.

**Figure:**
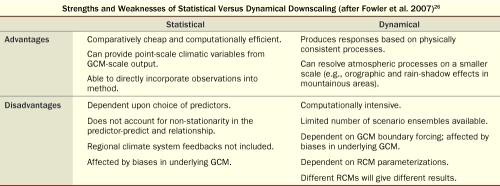
Strengths and Weaknesses of Statistical Versus Dynamical Downscaling (after Fowler et al. 2007)^26^

Wagener and colleagues are developing a framework to quantify the uncertainty in projections of water availability, a key area of concern for water managers, including thos.e at electric power plants. Coal, natural gas, and nuclear power plants all use water to cool plant operations, and climate change could have significant impacts on water resources.^23^ “In addition to providing a best estimate of future conditions, we will be able to quantify our confidence in this estimate,” Wagener says.

Richard Rood, a professor in the Atmospheric, Oceanic, and Space Sciences Department at the University of Michigan, is also working on accommodating uncertainty through the National Climate Predictions and Projections (NCPP) platform, a web-based program that offers decision makers guidance for interpreting modeling data and advice for putting uncertainties into context at a national scale.^24^ “When presented with the uncertainty associated with downscaled data, nearly one-half of the stakeholders working with the NCPP, including public health officials, say, ‘We are not really interested in the digital data. Can you provide us with descriptive data?’” Rood says.

But uncertainty is by no means a deal breaker. Wagener points to research showing that understanding the scope of uncertainty can help decision makers make sound choices.^25^ “If you give people information—for example, the weather forecast—and you tell them the most likely scenario or your best guess, and add the degree of confidence you have with that prediction, or add the uncertainty that goes with the prediction, it provides more information than if you don’t describe the uncertainty,” he says. “This leads to better decisions.”
